# Development and Validation of an *in-House* Library of Colombian *Candida auris* Strains with MALDI-TOF MS to Improve Yeast Identification

**DOI:** 10.3390/jof6020072

**Published:** 2020-05-27

**Authors:** Andrés Ceballos-Garzon, Daniela Amado, Norida Vélez, María José Jiménez-A, Crescencio Rodríguez, Claudia Marcela Parra-Giraldo

**Affiliations:** 1 Unidad de Proteómica y Micosis Humanas, Grupo de Enfermedades Infecciosas, Departamento de Microbiología, Facultad de Ciencias, Pontificia Universidad Javeriana, Bogotá D.C. 110231, Colombia; c-ceballos@javeriana.edu.co (A.C.-G.); d.amado@javeriana.edu.co (D.A.); velez.norida@javeriana.edu.co (N.V.); mjimenez.a@javeriana.edu.co (M.-J.J.-A.); 2 Grupo de Microbiología, Instituto Nacional de Salud, Bogotá 110931, Colombia; 3 Bruker Mexicana, Ciudad de México 01-16. Damas 130 Int.501 Col, San José Insurgentes, Ciudad de México 03900, Mexico; crescencio.rodriguez@bruker.com

**Keywords:** *Candida auris*, yeast identification, library construction, MALDI-TOF MS

## Abstract

Background: *Candida auris* is characterized for having a high genetic variability among species. MALDI-TOF MS library contains spectra from only three strains of *C. auris*, which makes difficult the identification process and gives low scores at the species level. Our aim was to construct and validate an internal library to improve *C. auris* identification with Colombian clinical strains. Methods: From 30 clinical strains, 770 mass spectra were obtained for the construction of the database. The validation was performed with 300 strains to compare the identification results in the BDAL and *C. auris* Colombia libraries. Results: Our library allowed a complete, 100% identification of the evaluated strains and a significant improvement in the scores obtained, showing a better performance compared to the Bruker BDAL library. Conclusions: The strengthening of the database is a great opportunity to improve the scoring and *C. auris* identification. Library data are available via ProteomeXchange with identifier PXD016387.

## 1. Introduction

Invasive candidiasis is the most common fungal disease in hospitalized patients, with more than 50,000 deaths worldwide [1]. The main etiological agent is *Candida albicans*. However, the prevalence of non-albicans species (e.g., *C. glabrata, C. parapsilosis, C. tropicalis, C. krusei, C auris*) is increasing [2,3].

*C. auris* is a multi-drug resistant yeast (MDR), which was first recovered in 2009 from the external ear canal of a 70-year-old female Japanese patient in Tokyo [4]. Henceforth, it has been reported in over 20 countries on five continents, including Colombia, with reports made by our group [5,6]. *C. auris* candidemia is associated with mortality rates of about 30–60%, depending on the setting [7]. 

The identification of *C. auris* is a challenge. Only a few phenotypic methods detect this species correctly. It is often classified mistakenly as *C. haemulonii*, *C. famata*, *C. lusitaniae*, *and C. parapsilosis* by VITEK 2; as *R. glutinis by* API 20C AUX systems; as *C. catenulate* and *C.* haemulonii by BD Phoenix, and as *C. guilliermondii* and *C. tropicalis* by MicroScan [5,7]. The unique biochemistry methodology that is capable to detect it with a high reliability is the version *VITEX 2 XL* (bioMérieux version 8.01). However, specialized techniques, such as DNA sequencing and matrix-assisted laser desorption ionization-time of flight mass spectrometry (MALDI-TOF MS) are the most efficient methodologies for a reliable identification [8,9,10].

In the last decade, MALDI-TOF MS has been proven as a solid option for microbial identification through the comparison of its profiles of cytoplasmic protein spectra. This technique provides faster identification than traditional molecular methods, and its usefulness has been evaluated and used in many taxonomic groups, including fungi [11].

The capabilities of this method depend mainly on the content of the protein spectral libraries that are used. Currently, the identification of *C. auris* by MALDI-TOF MS Biotyper OC 3.1.66 BRUKER-BDAL contains spectra of only three *C. auris* strains (CBS KCTC 17809, CBS KCTC 17810 from Korea) and *C. auris* (DSM 21092T CBS from Japan), which made it difficult to obtain a high identification score in our experience with Colombians strains. However, Vatanshenassan et al. published good scores obtained in the identification of 50 *C. auris* strains from United States, Netherlands, India, Israel, and South Africa using Bruker MBT Compass Library, Revision E MBT 7854 [12,13]. 

In 2018, Escandón et al. performed a phylogenetic analysis based on the sequencing of the complete genome of *C. auris* Colombian strains. They made a comparison with countries such as South Africa, Japan, Pakistan, and India, and observed polymorphism variants of nucleotides with 140 Venezuelan strains. This indicated the great difference that exists in each geographic region of *C. auris* strains [14,15]. It is important to create a library with strains from different geographical locations, which will make it possible to strengthen libraries for greater and better identification, as well as to facilitate epidemiological studies and clinical diagnoses. Herein, we constructed and validated an internal library for the identification of *C. auris* with Colombian clinical strains.

## 2. Methods

### 2.1. Ethics’ Statement

The authors confirm that the ethical policies of the journal, as noted on the journal’s author guidelines page, have been adhered to. In this investigation, no ethical approval was required. The data used were strains spectra obtained by MALDI-TOF MS. All patients are anonymized and only the code of strains was transferred for this investigation. Therefore, no informed consent was required.

### 2.2. Characteristics of Strains Used to Make the in-House Library

The library was created with 30 Colombian clinical strains: The ones that were used to make the first reports of *C. auris* in Colombia, from the outbreak reported in the north and center of the country previously identified as a *C. auris* by MALDI-TOF MS [5,6].

### 2.3. Construction of In-House Library “C. auris Colombia”

Strains preserved in our bank were cultured from glycerol stocks stored at −80 °C, after incubation of strains at 35 °C for 24–36 h on Sabouraud dextrose agar (SDA). Protein extraction was performed using the protein extraction in formic acid/ethanol method, according to the Bruker Daltonics’ protocol with minor modifications as reported by Marklein in 2009 [16]. Briefly, two or three colonies were mixed with 300 μL of HPLC-grade water until homogenization and then 900 μL of 100% ethanol (Sigma-Aldrich, St Louis, MO) were added. After centrifugation at 15,000 g for 2 minutes, the pellet was dried at 25 °C, reconstituted in equal volumes of 100% formic acid (Sigma- Aldrich) and acetonitrile (Sigma-Aldrich) (20 µL each), mixed thoroughly, and centrifuged at 15,000 g for 2 min. One microliter of supernatant was spotted onto a 96-spot steel plate (Bruker Daltonic) and left to dry at room temperature before the addition of 1 µL of the α-Cyano-4-hydroxycinnamic acid HCCA matrix (provided by the supplier). For yeast identification, each sample was tested in duplicate. Only the spot reporting the highest probability score of identification was considered [17]. MALDI-TOF MS mass range for measuring spectra was made between 2000 and 20,000 Daltons, results were then compared, and a score was obtained according to the manufacturer’s technical specifications, as follows: Highly probable species identification (≥2300–3000), secure genus identification, probable species identification (≥2000–2299), probable genus identification (1700–1999), and not reliable identification (<1700).

For the library construction, each protein extract from strains was placed on 10 different spots on the plate to generate mass spectra data using FlexControl version 3.4. Only higher quality spectra (low noise, recognizable peaks, and more than 10^4^ units of intensity) analyzed with FlexAnalysis software were considered for the creation of combined Main Spectra (MSP) library with a minimum of 20 spectra per strain. Compound spectra were created for the library using the “MSP creation” function of the MALDI Biotyper version 3.1 7311 reference spectra (main spectra) (Bruker Daltonics, Bremen, Germany), a library named “*Candida auris* Colombia”. For phylogenetic analysis of the mass spectra of *C. auris*, the dendrogram was generated using the respective functionality of the MALDI-TOF MS Biotyper 3.1 7311 offline client. The mass spectra of 30 respective strains with a score value of >2 was considered for the preparation of the dendrogram. The spectra of all strains tested were analyzed as a core-oriented dendrogram using an arbitrary distance level of 1000 as the cut-off [18,19,20].

The mass spectrometry proteomics data were deposited into the ProteomeXchange Consortium via the PRIDE [21] partner repository with the dataset identifier PXD016387.

### 2.4. Validation of Colombia Candida auris Library

**Figure 1:**
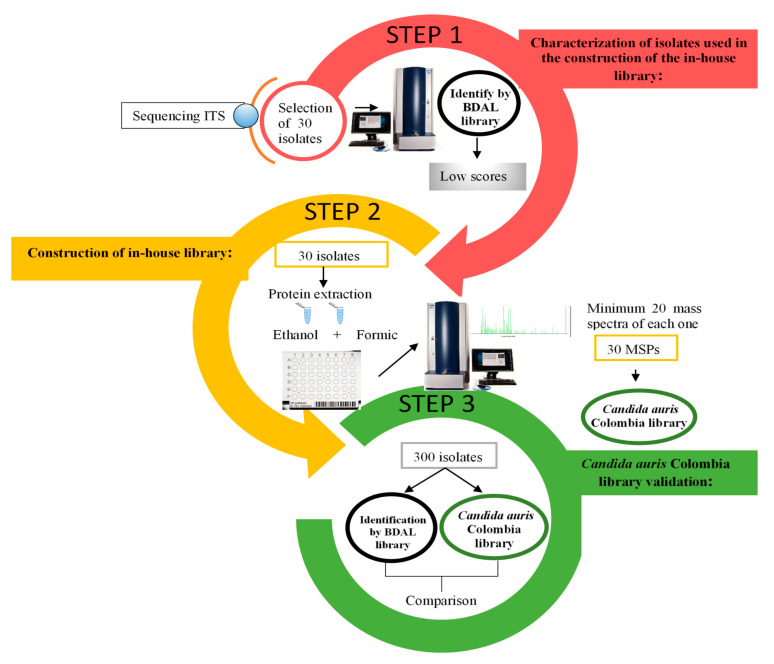
Flowchart methodology of the library construction and validation.

The library evaluation was effectuated with 300 *C. auris* strains from different national institutions that were identified in our laboratory. The spectra obtained for these identifications were used to evaluate our library [22], following the same protein extraction protocol cited before, comparing results between BDAL (Bruker) and *Candida auris* Colombia (*in house*) libraries (Figure 1). Potential false positives from non-*C. auris* strains, such as *C. catenulata*, *C. duobushaemulonii*, *C. famata*, *C. guilliermondii*, *C. haemulonii*, *C. krusei*, *C. lusitaniae* and *C. parapsilosis*, were evaluated. None of the yeasts was misidentified.

## 3. Results

### 3.1. Construction of the in-House Library “C. auris Colombia”

**Figure 2:**
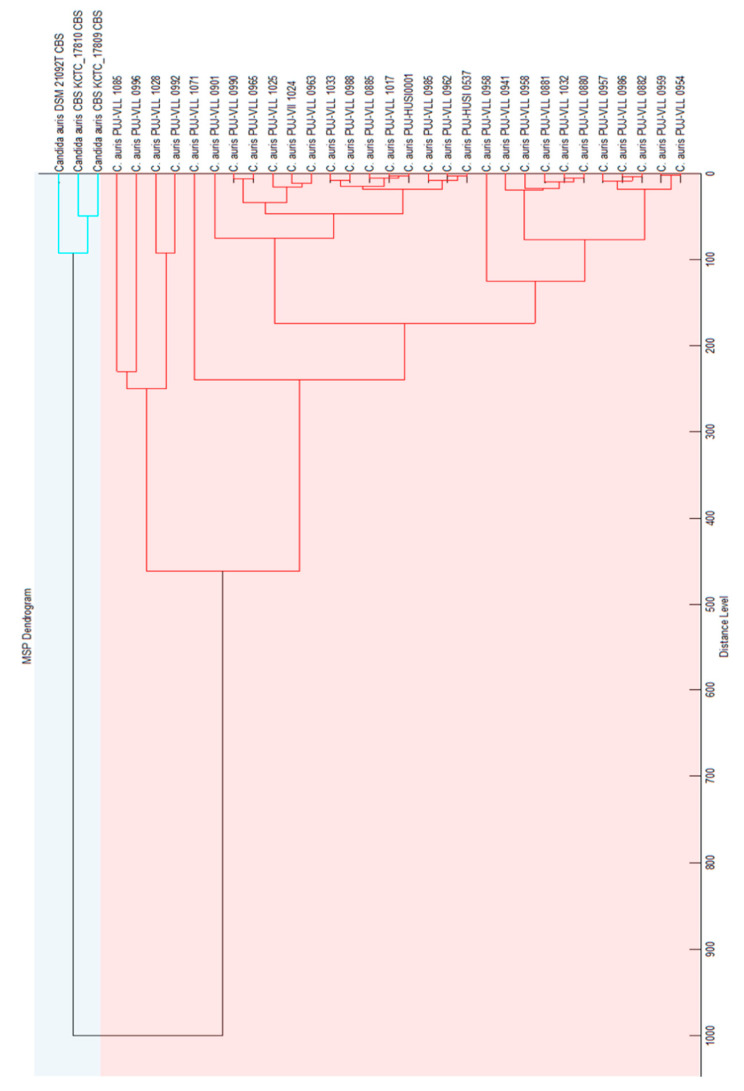
*C. auris* BDAL library (three strains) and *C. auris* Colombia library (30 strains) dendrogram.

**Figure 3:**
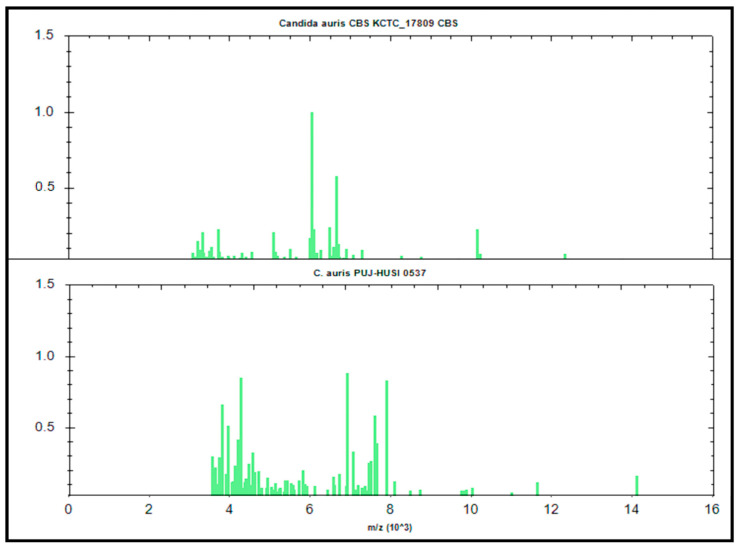
*C. auris* MSP from BDAL library (upper) and another one selected MSP from Colombian strains (lower).

The MSPs for the *in-house* library construction was generated through the accumulation of 777 mass spectra from 30 *C. auris* clinical strains (Figure S1). Subsequently, the three MSPs from *C. auris* in the BDAL library and our 30 MSPs were used for the dendrogram construction (Figure 2). We observed that our MSPs were different from the BDAL MSPs, which led the identification of *C. auris* Colombian strains to present scores of <2.0 in the BDAL library. The dendrogram obtained showed two clades (Colombian-Oriental strains) when compared to the mass spectrum of each isolate. This library is available for everyone and those interested only need to write to the corresponding author; validating with strains from other geographic areas is important for us. The *C. auris* Colombia library showed a greater MSP diversity compared with BDAL (one representative MSP) (Figure 3).

### 3.2. Candida auris Colombia Library Validation

With the BDAL library, 199/300 strains (66%) were identified, belonging to four distinct identification levels: Highly probable species identification (0/300); secure genus, probable species identification (62/300); probable genus identification (137/300); and not reliable identification (101/300).

**Figure 4:**
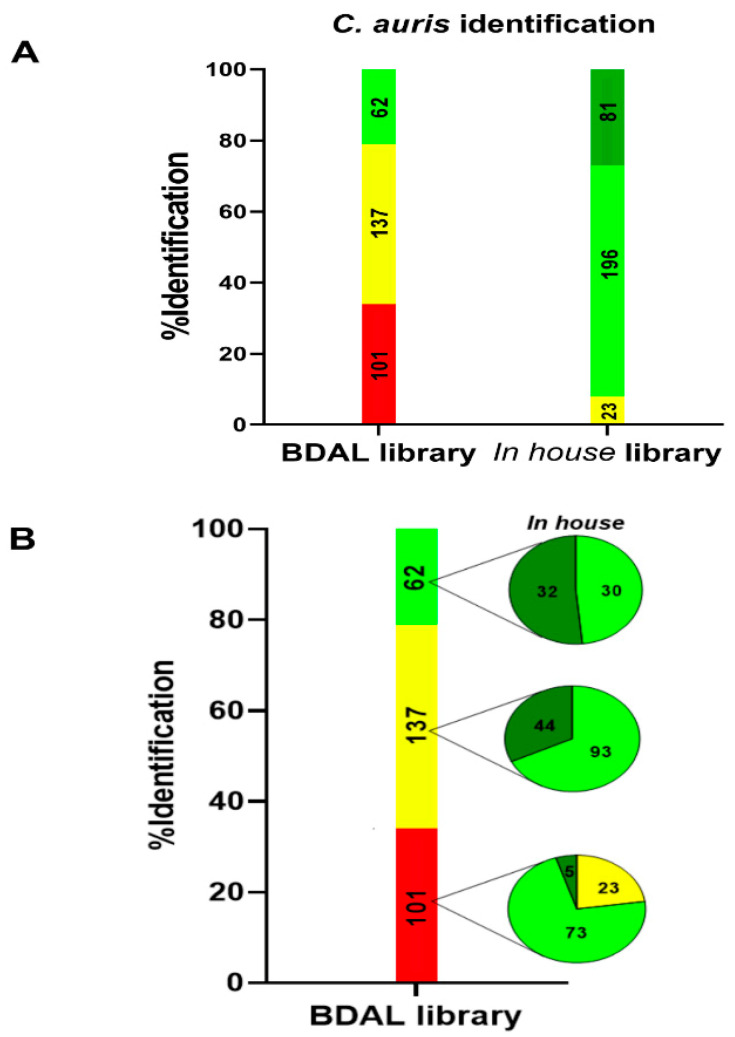
BDAL and *Candida auris* Colombia libraries’ identification results. (**A**) Total results independently obtained from each library. (**B**) Yeast comparison identification from BDAL and Colombia databases. Pie charts represent the score of the identifications of 300 strains by Colombia in-house library inside the scores obtained in the BDAL library. The MALDI-TOF MS results, according to the manufacturer’s technical specifications, were as follows: Highly probable species identification (>2300), green; secure genus, probable species identification (≥2000), light green; probable genus (1700–2000), yellow; and no reliable identification (<1700), red.

When performing MALDI-TOF MS identification with the *Candia auris* Colombia library, better results were obtained, identifying the totality of the isolations and improving the identification score 300/300 (100%). Strains were identified belonging to three distinct identification levels: Highly probable species identification (81/300); secure genus, probable species identification (196/300); and probable genus identification (23/300) (Figure 4).

## 4. Discussion

Since the appearance of *C. auris* in the world in 2009 [4], the field of medical mycology has been revolutionized. *C. auris* is a yeast that not only presents an atypical susceptibility profile but also a laborious identification [10]. Direct protein analysis using MALDI-TOF MS offers a great opportunity to identify microorganisms that are difficult or impossible to detect with the biochemical methods [23]. In this study, we constructed a *C. auris* library to improve the identification of the yeasts due to the lack of MSPs in the BDAL library. When comparing the mass spectra of BDAL with the *C. auris* Colombia library, we observed that the latter had significant differences in the spectra. This probably occurred due to protein extraction protocol or the geographical origin of these isolations, which is consistent with Escandón et al., who observed a phylogenetic difference between Colombian strains and those from other countries [14]. This distinction also occurs with phenotypic characteristics, such as a strain’s susceptibility, where the resistance for antifungal molecules is different depending on the geographic area [24]. Also, resistance mechanisms varied in each country. A multicentric study stated that the mutations in *ERG11* implicated in azole resistance varied according to the geographic region of the strains [25]. The variation in the characteristics of the different strains around the world made the identification with traditional methods difficult, even with MALDI-TOF MS. Therefore, when we identified our strains using MALDI-TOF MS with BDAL library, the score of identification was <2 and when we built the dendrogram with our strains’ spectra and BDAL strain spectra, we observed a deep separation that was represented in the clear division between the Colombian and the Eastern strains.

*Candida auris* Colombia library presented multiple spectra of *C. auris* that improved the identification scores in 100% of the strains used for the validation of our library. This confirmed that a greater diversity and the robustness of the database improved the identification accuracy of *C. auris* based on MSPs in MALDI-TOF MS.

We know that a weakness of our study was not having *C. auris* strains from the four phylogenetic clades, but as the CDC says, “supplemental MALDI-TOF databases that include additional *C. auris* strains from all four of the phylogenetic clades may enable users to overcome identification challenges by providing consistently higher MALDI identification scores. MicrobeNet is one example of a free online MALDI database of rare and unusual pathogens that is curated by CDC experts [8].” With this Colombia library, we want to provide spectra from South American strains (clade IV) and contribute to the construction of a robust database of all clades.

## 5. Conclusions

The Bruker’s library version 3.1 7311 reference spectra (main spectra) had only three *C. auris* strains from Korea and Japan, which entailed identification problems and a low identification score. Our database had a remarkable improvement in the identification of the 300 strains, evidencing that the strengthening of the database is a great opportunity to improve the scoring and the identification of *C. auris*. We are interested in sharing this library and contributing to the construction of a robust database.

## Data Availability

The datasets generated and analyzed during the current study are available via ProteomeXchange with identifier PXD016387.

## Supplementary Materials

The following are available online at https://www.mdpi.com/2309-608X/6/2/72/s1, Figure S1: Mass Spectra from the strains used in Colombia library creation.

## Abbreviations

**MDR**	Multidrug resistant
**MALDI-TOF MS**	Matrix Assisted Laser Desorption and Ionization-Time of Flight Mass Spectrometry
**MSP**	Main spectra library
**HCCA**	α-Cyano-4-hydroxycinnamic acid

## Footnote

Part of this study was accepted at the Trends in Medical Mycology Congress TIMM-9, 11–14 October 2019, in Nice (France), as a poster session. Abstract #559.

## References

[1] Kullberg B.J., Arendrup M.C. (2015). Invasive Candidiasis. N. Engl. J. Med..

[2] Lamoth F., Lockhart S.R., Berkow E.L., Calandra T. (2018). Changes in the epidemiological landscape of invasive candidiasis. J. Antimicrob. Chemother..

[3] Yapar N. (2014). Epidemiology and risk factors for invasive candidiasis. Ther. Clin. Risk Manag..

[4] Satoh K., Makimura K., Hasumi Y., Nishiyama Y., Uchida K., Yamaguchi H. (2009). Candida auris sp. nov., a novel ascomycetous yeast isolated from the external ear canal of an inpatient in a Japanese hospital. Microbiol. Immunol..

[5] Morales-López S.E., Parra-Giraldo C.M., Ceballos-Garzón A., Martínez H.P., Rodríguez G.J., Álvarez-Moreno C.A., Rodríguez J.Y. (2017). Invasive Infections with Multidrug-Resistant Yeast Candida auris, Colombia. Emerg. Infect. Dis..

[6] Parra-Giraldo C.M., Valderrama S.L., Cortes-Fraile G., Garzón J.R., Ariza B.E., Morio F., Linares-Linares M.Y., Ceballos-Garzón A., de la Hoz A., Hernandez C. (2018). First report of sporadic cases of Candida auris in Colombia. Int. J. Infect. Dis..

[7] Forsberg K., Woodworth K., Walters M., Berkow E.L., Jackson B., Chiller T., Vallabhaneni S. (2019). *Candida auris*: The recent emergence of a multidrug-resistant fungal pathogen. Med. Mycol..

[8] Centers for Disease Control and Prevention Identification of Candida auris-Fungal Diseases. https://www.cdc.gov/fungal/candida-auris/recommendations.html.

[9] Kordalewska M., Zhao Y., Lockhart S.R., Chowdhary A., Berrio I., Perlin D.S. (2017). Rapid and accurate molecular identification of the emerging multidrug-resistant pathogen Candida auris. J. Clin. Microbiol..

[10] Ceballos-Garzón A., Cortes G., Morio F., Zamora-Cruz E.L., Linares M.Y., Ariza B.E., Valderrama S.L., Garzón J.R., Alvarez-Moreno C.A., Le Pape P. (2019). Comparison between MALDI-TOF MS and MicroScan in the identification of emerging and multidrug resistant yeasts in a fourth-level hospital in Bogotá, Colombia. BMC Microbiol..

[11] Panda A., Ghosh A.K., Mirdha B.R., Xess I., Paul S., Samantaray J.C., Srinivasan A., Khalil S., Rastogi N., Dabas Y. (2015). MALDI-TOF mass spectrometry for rapid identification of clinical fungal isolates based on ribosomal protein biomarkers. J. Microbiol. Methods.

[12] Kathuria S., Singh P.K., Sharma C., Prakash A., Masih A., Kumar A., Meis J.F., Chowdhary A. (2015). Multidrug-Resistant Candida auris Misidentified as Candida haemulonii. J. Clin. Microbiol..

[13] Vatanshenassan M., Boekhout T., Meis J.F., Berman J., Chowdhary A., Ben-Ami R., Sparbier K., Kostrzewa M. (2019). Candida auris Identification and Rapid Antifungal Susceptibility Testing Against Echinocandins by MALDI-TOF MS. Front. Cell. Infect. Microbiol..

[14] Escandón P., Chow N.A., Caceres D.H., Gade L., Berkow E.L., Armstrong P., Rivera S., Misas E., Duarte C., Moulton-Meissner H. (2018). Molecular Epidemiology of Candida auris in Colombia Reveals a Highly Related, Countrywide Colonization With Regional Patterns in Amphotericin B Resistance. Clin. Infect. Dis..

[15] Rhodes J., Fisher M.C. (2019). Global epidemiology of emerging Candida auris. Curr. Opin. Microbiol..

[16] Marklein G., Josten M., Klanke U., Müller E., Horré R., Maier T., Wenzel T., Kostrzewa M., Bierbaum G., Hoerauf A. (2009). Matrix-assisted laser desorption ionization-time of flight mass spectrometry for fast and reliable identification of clinical yeast isolates. J. Clin. Microbiol..

[17] Pinto A., Halliday C., Zahra M., van Hal S., Olma T., Maszewska K., Iredell J.R., Meyer W., Chen S.C.A. (2011). Matrix-assisted laser desorption ionization-time of flight mass spectrometry identification of yeasts is contingent on robust reference spectra. PLoS ONE.

[18] Kostrzewa M., Schubert S. (2016). MALDI-TOF Mass Spectrometry in Microbiology. British Library Cataloguing in Publication Data.

[19] Yaman G., Akyar I., Can S. (2012). Evaluation of the MALDI TOF-MS method for identification of Candida strains isolated from blood cultures. Diagn. Microbiol. Infect. Dis..

[20] Rodríguez-Leguizamón G., Fiori A., López L.F., Gómez B.L., Parra-Giraldo C.M., Gómez-López A., Suárez C.F., Ceballos A., Van Dijck P., Patarroyo M.A. (2015). Characterising atypical Candida albicans clinical isolates from six third-level hospitals in Bogotá, Colombia. BMC Microbiol..

[21] Perez-Riverol Y., Csordas A., Bai J., Bernal-Llinares M., Hewapathirana S., Kundu D.J., Inuganti A., Griss J., Mayer G., Eisenacher M. (2019). The PRIDE database and related tools and resources in 2019: Improving support for quantification data. Nucleic Acids Res..

[22] Armstrong P.A., Rivera S.M., Escandon P., Caceres D.H., Chow N., Stuckey M.J., Díaz J., Gomez A., Vélez N., Espinosa-Bode A. (2019). Hospital-Associated Multicenter Outbreak of Emerging Fungus *Candida auris*, Colombia, 2016. Emerg. Infect. Dis..

[23] Singhal N., Kumar M., Kanaujia P.K., Virdi J.S. (2015). MALDI-TOF mass spectrometry: An emerging technology for microbial identification and diagnosis. Front. Microbiol..

[24] Chowdhary A., Prakash A., Sharma C., Kordalewska M., Kumar A., Sarma S., Tarai B., Singh A., Upadhyaya G., Upadhyay S. (2018). A multicentre study of antifungal susceptibility patterns among 350 Candida auris isolates (2009–17) in India: Role of the ERG11 and FKS1 genes in azole and echinocandin resistance. J. Antimicrob. Chemother..

[25] Lockhart S.R., Etienne K.A., Vallabhaneni S., Farooqi J., Chowdhary A., Govender N.P., Colombo A.L., Calvo B., Cuomo C.A., Desjardins C.A. (2017). Simultaneous Emergence of Multidrug-Resistant *Candida auris* on 3 Continents Confirmed by Whole-Genome Sequencing and Epidemiological Analyses. Clin. Infect. Dis..

