# Supplementary figures and images for: Development and Validation of an in-House Library of Colombian Candida auris Strains with MALDI-TOF MS to Improve Yeast Identification
Source: J Fungi (Basel). 2020 May 27;6(2):72. doi: 10.3390/jof6020072 (PMC7344545; doi:10.3390/jof6020072)

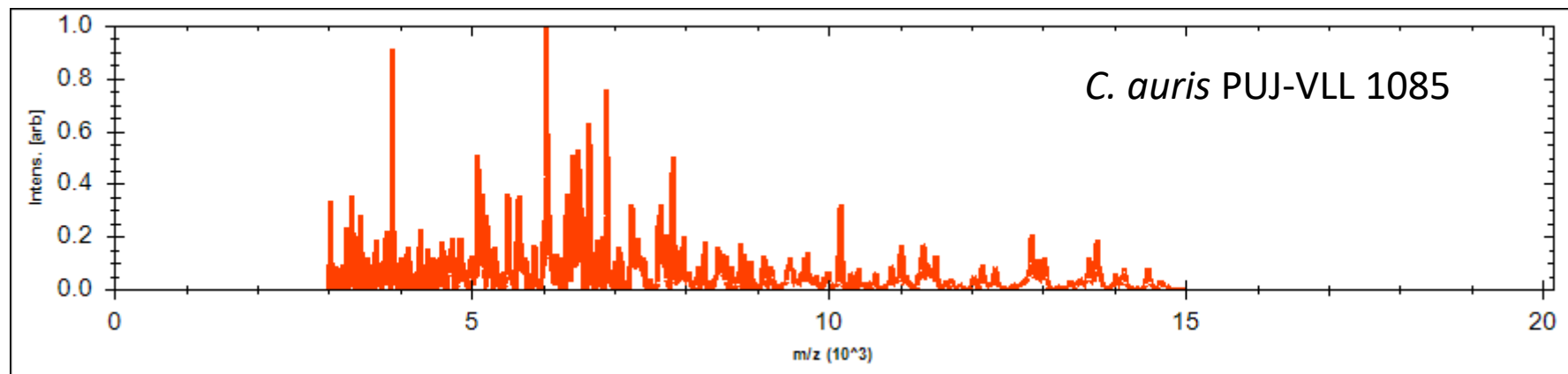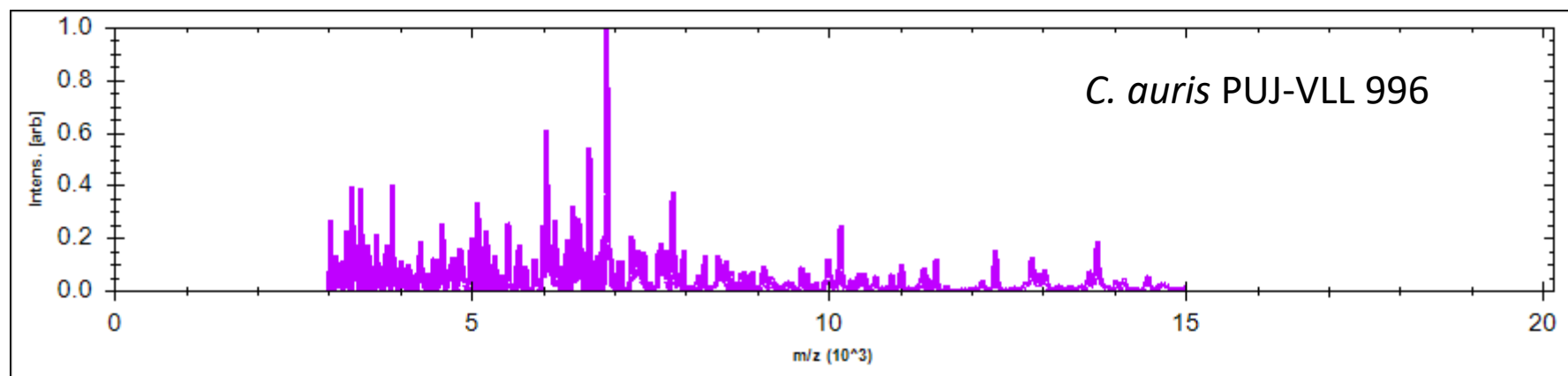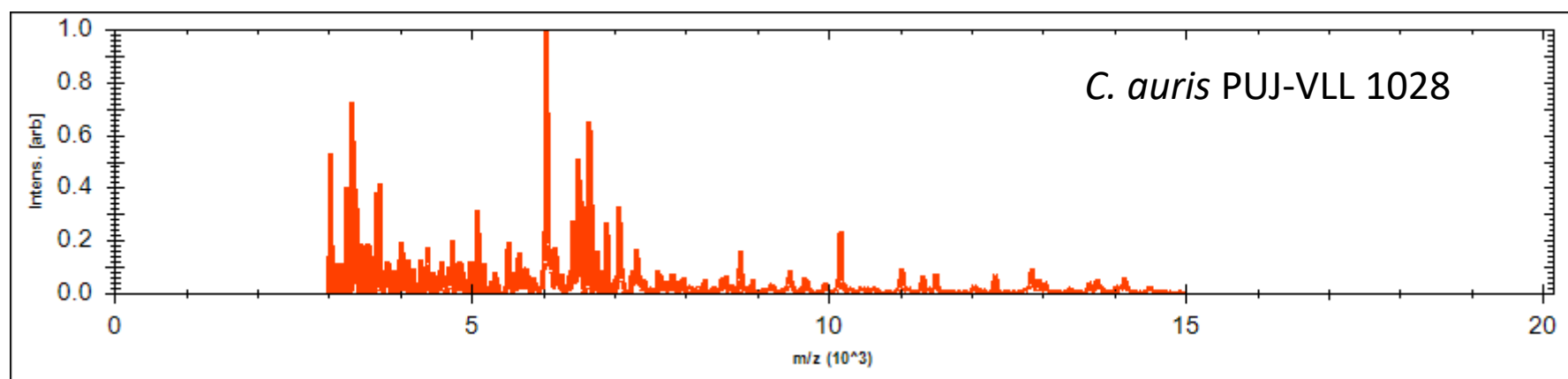

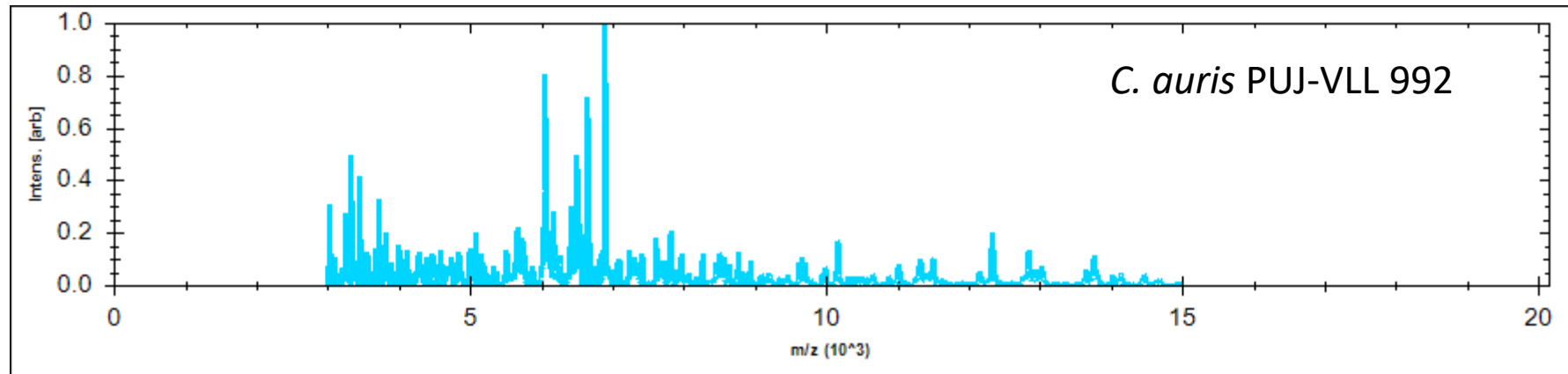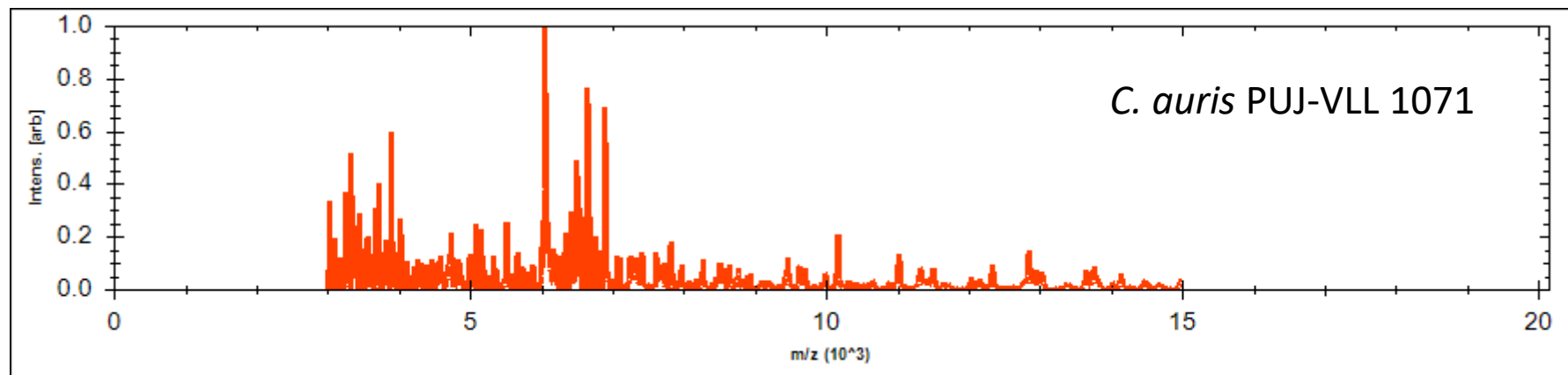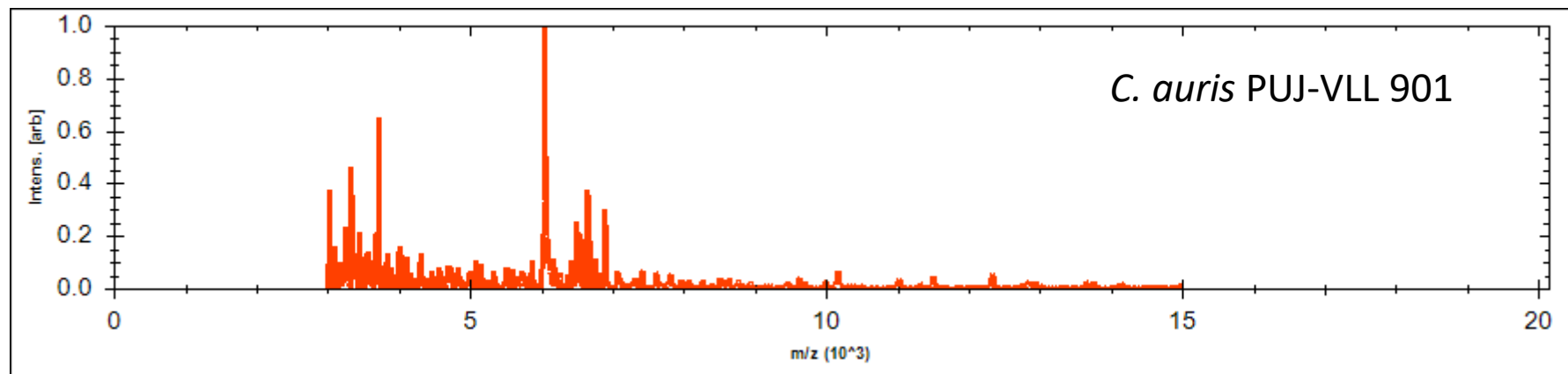

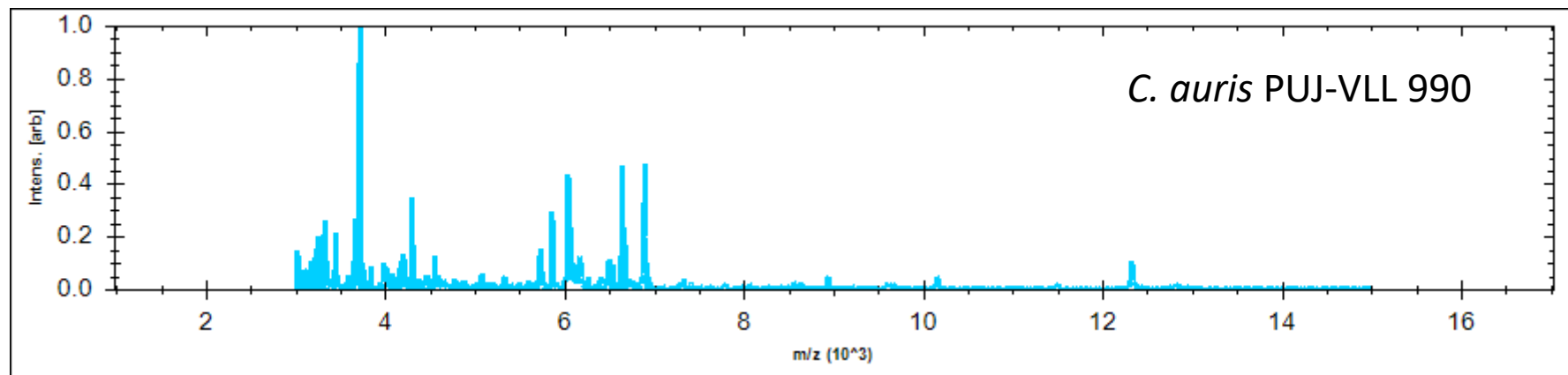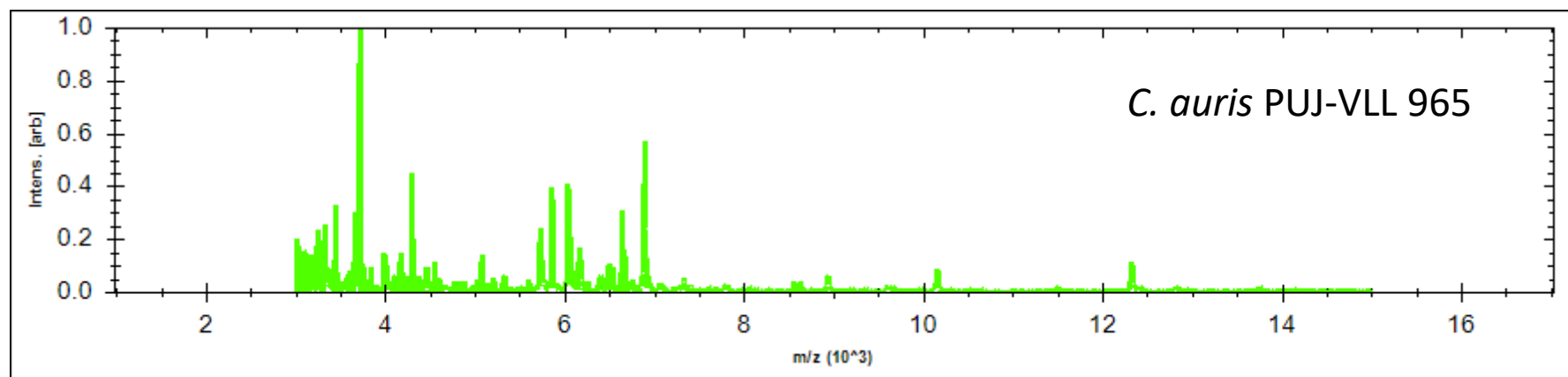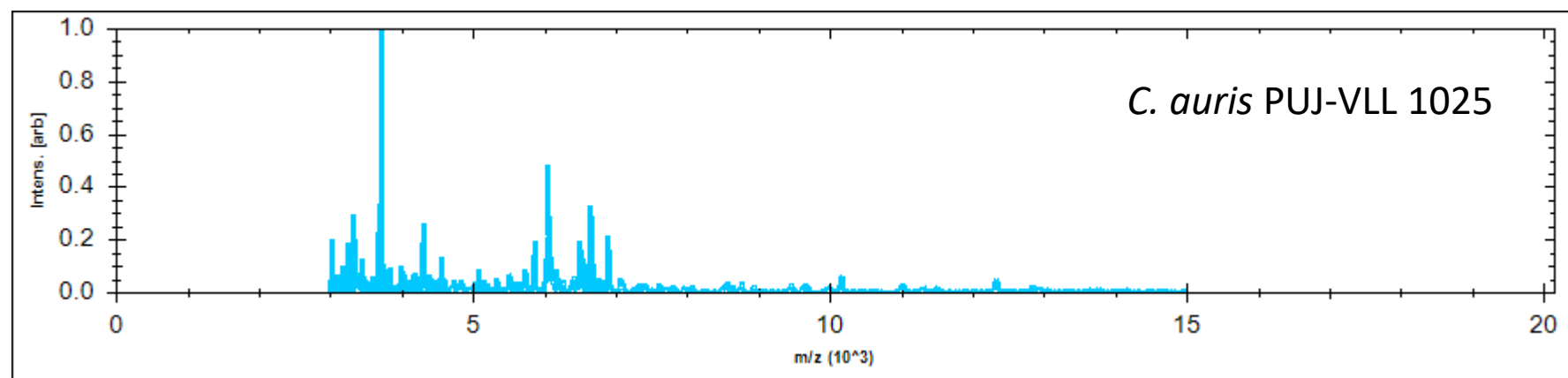

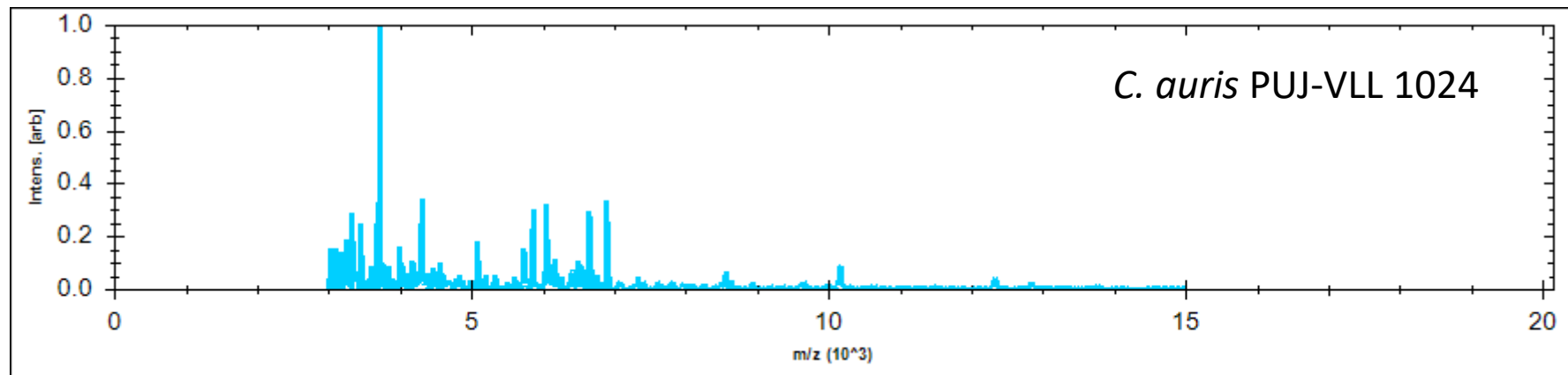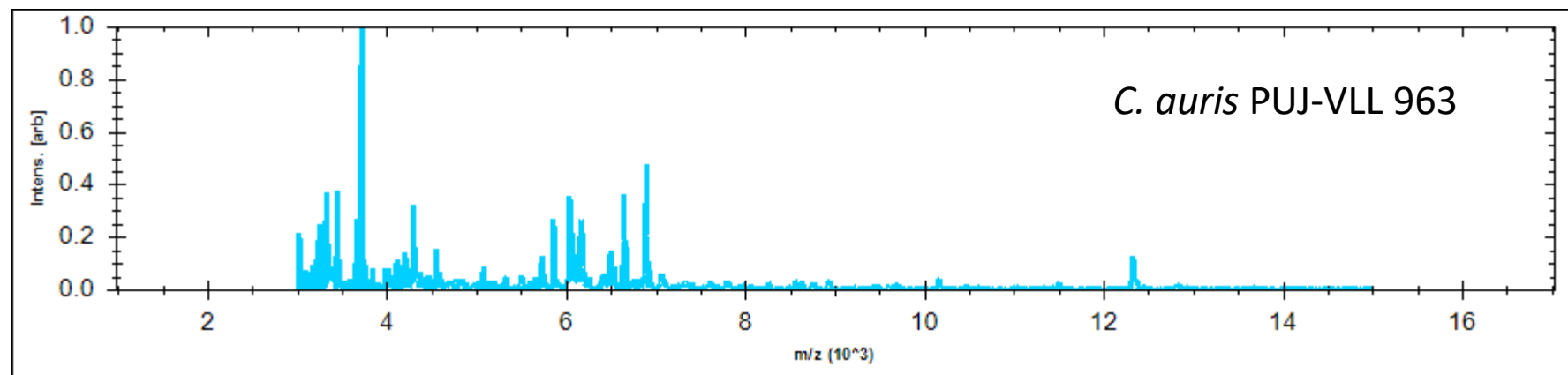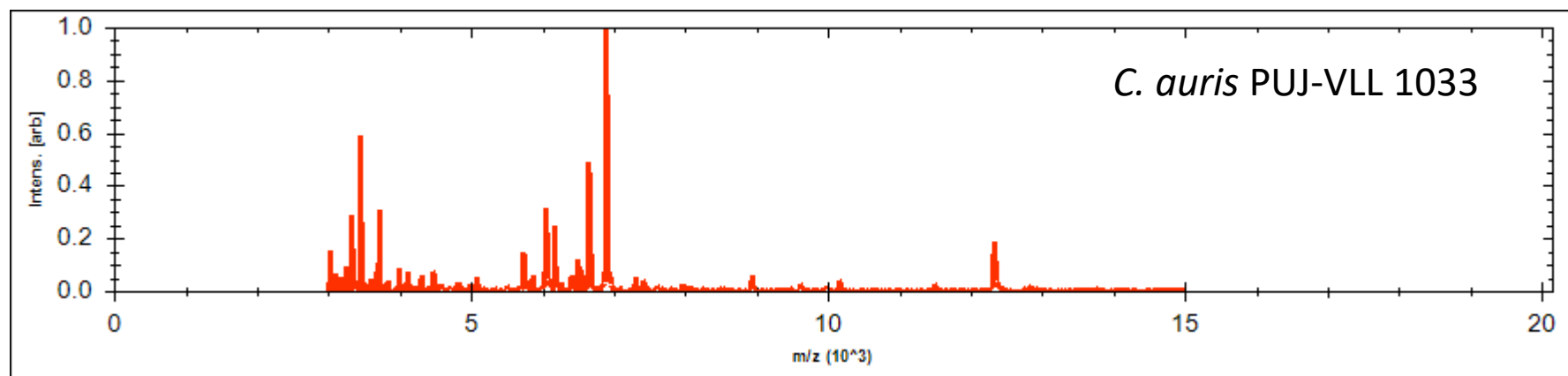

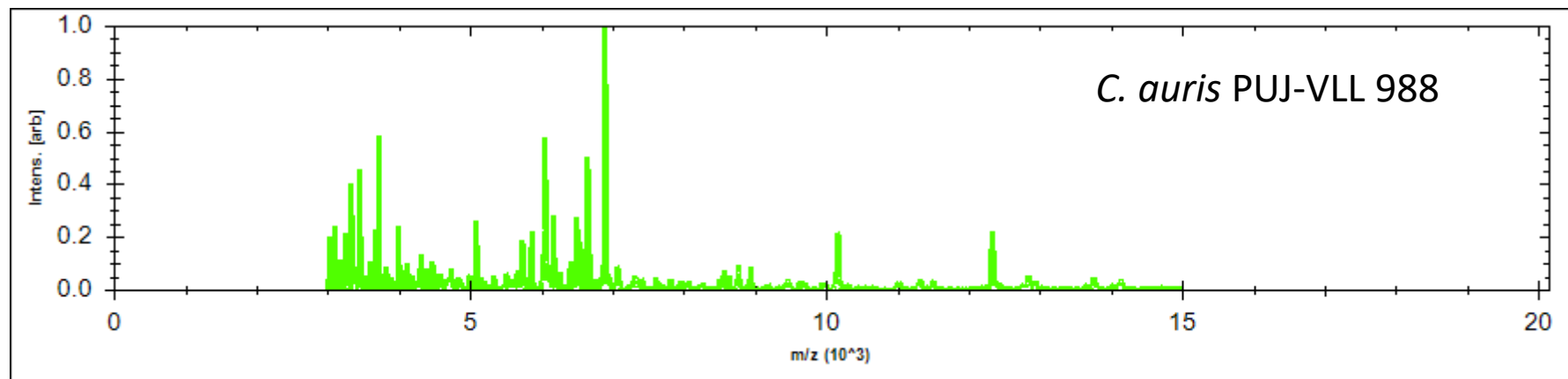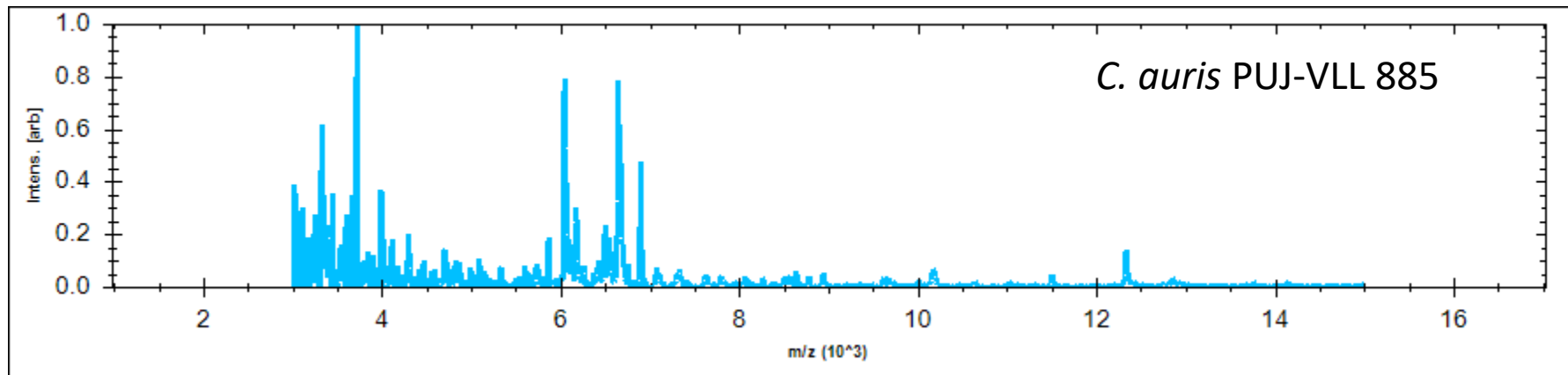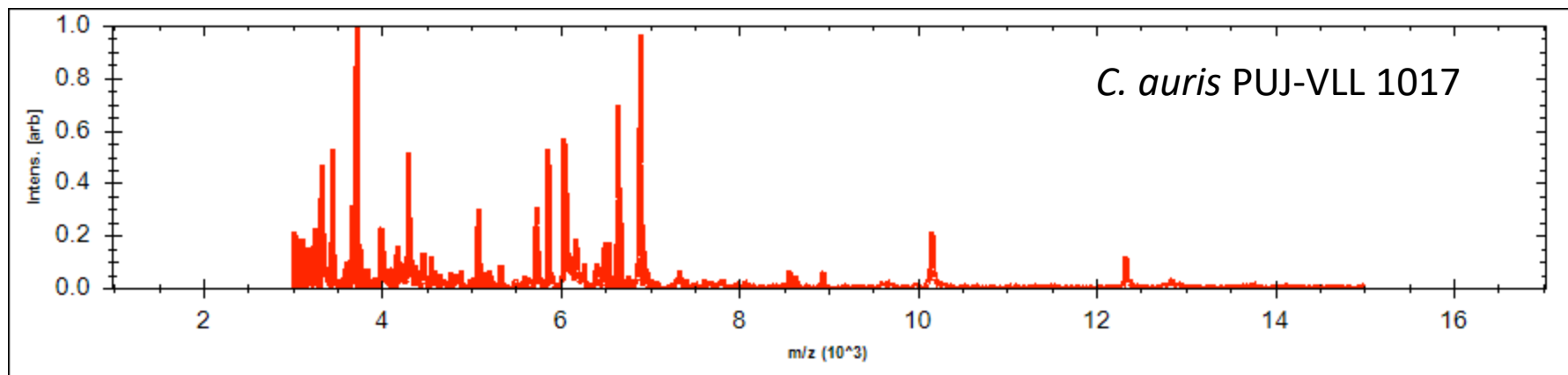

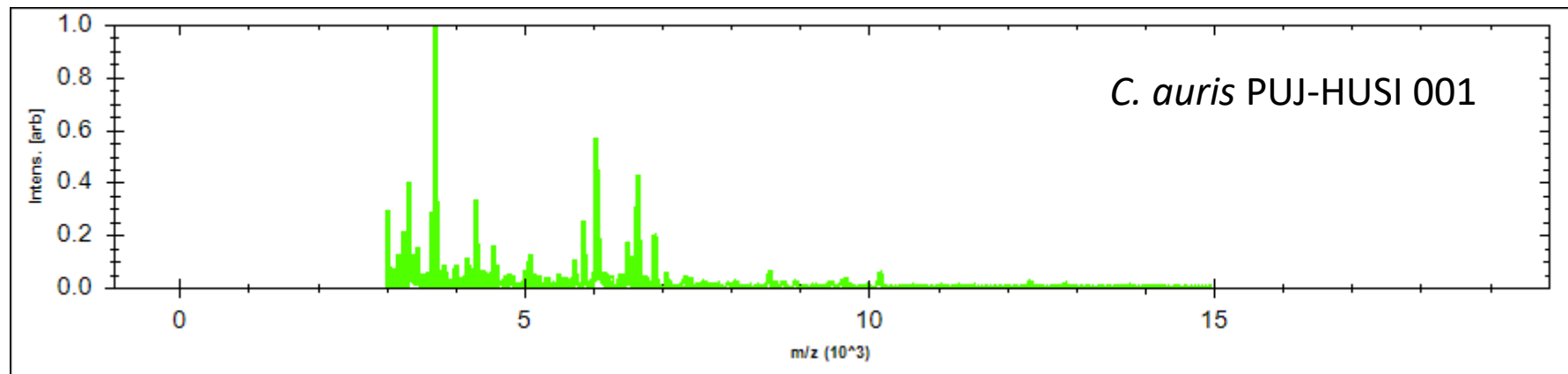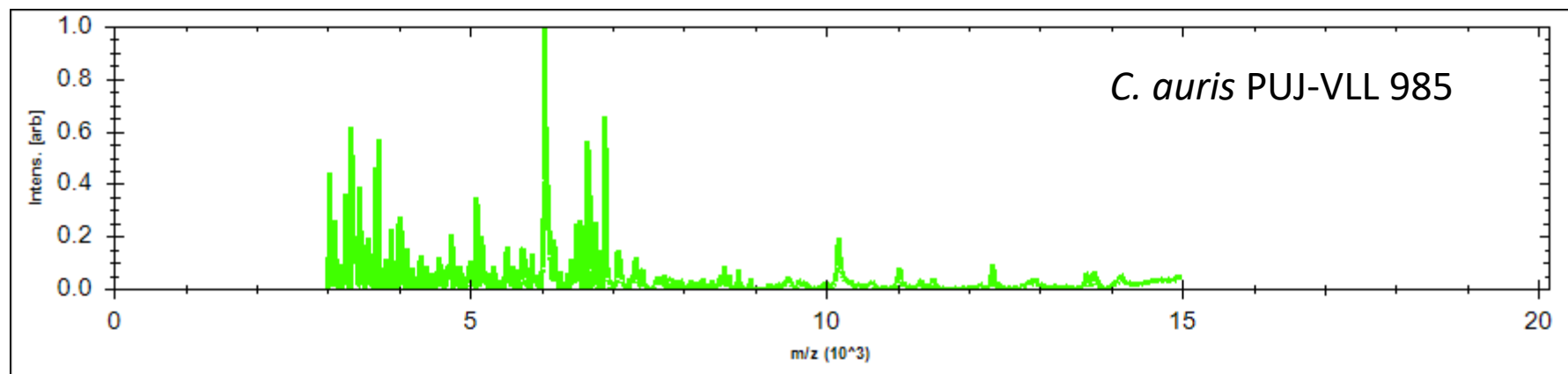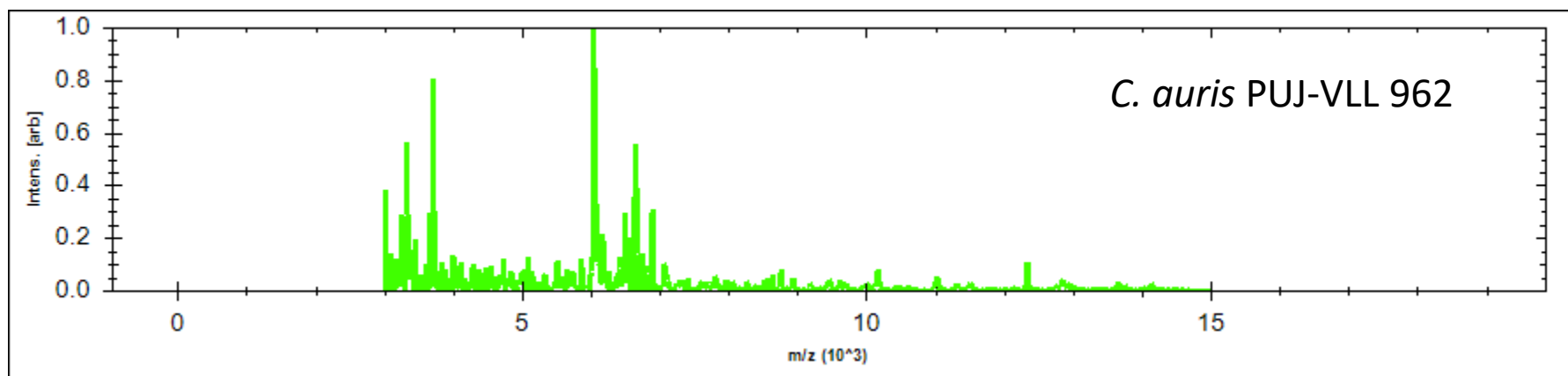

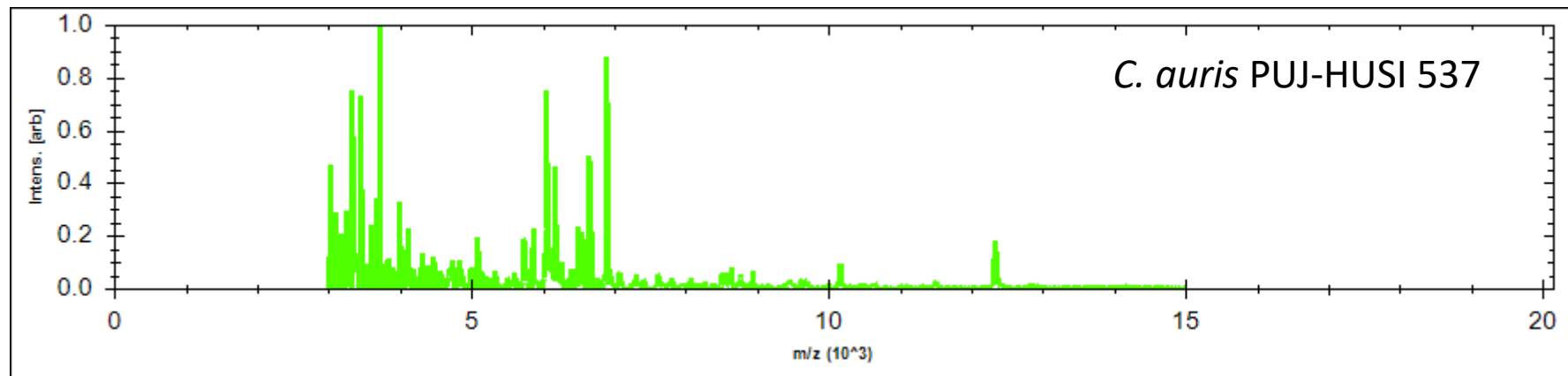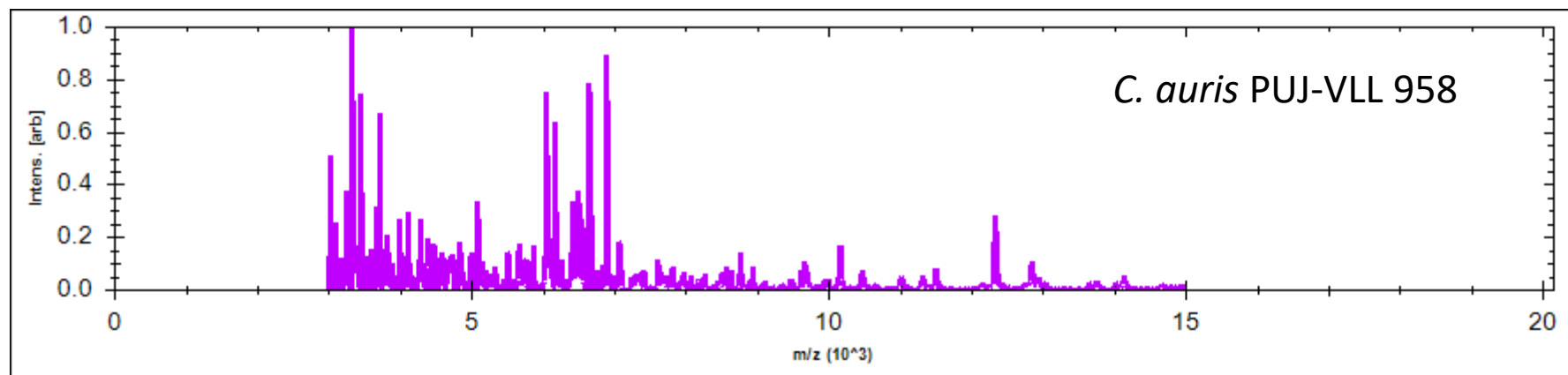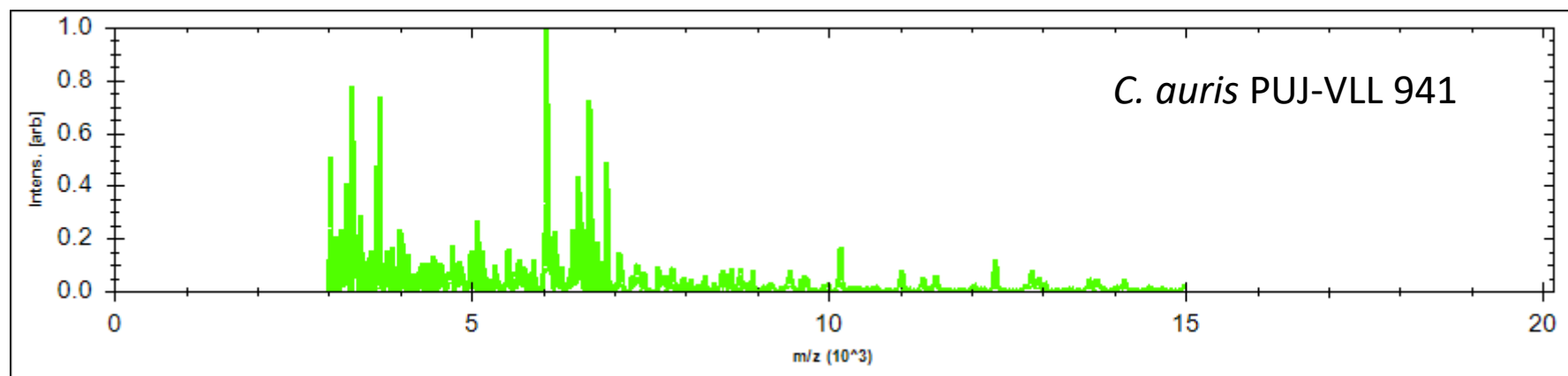

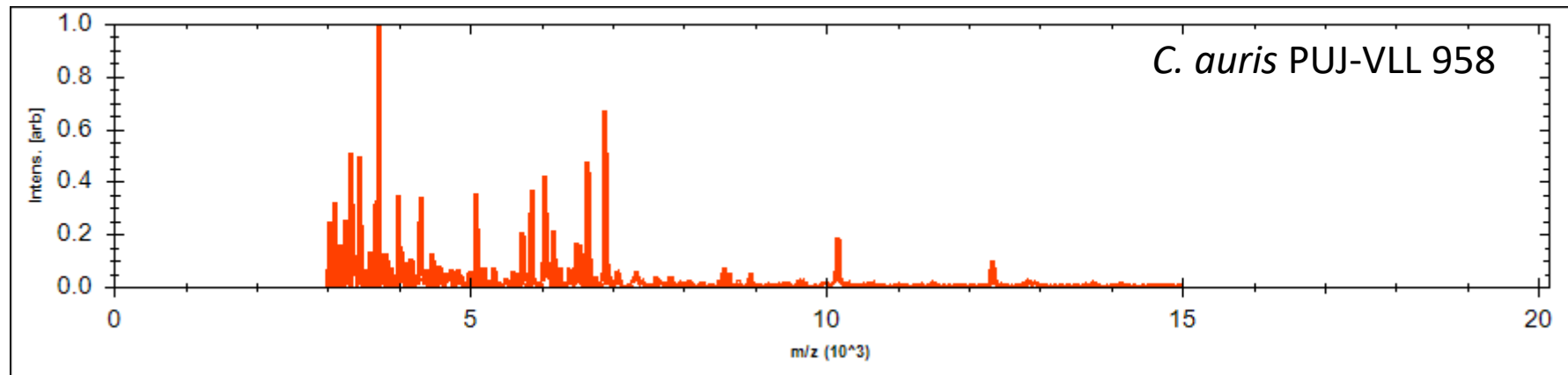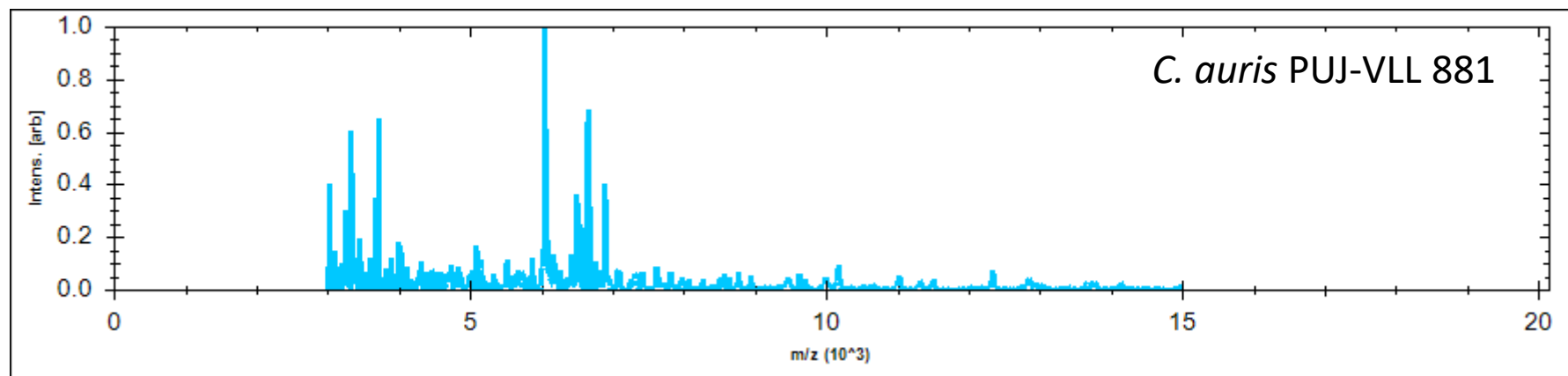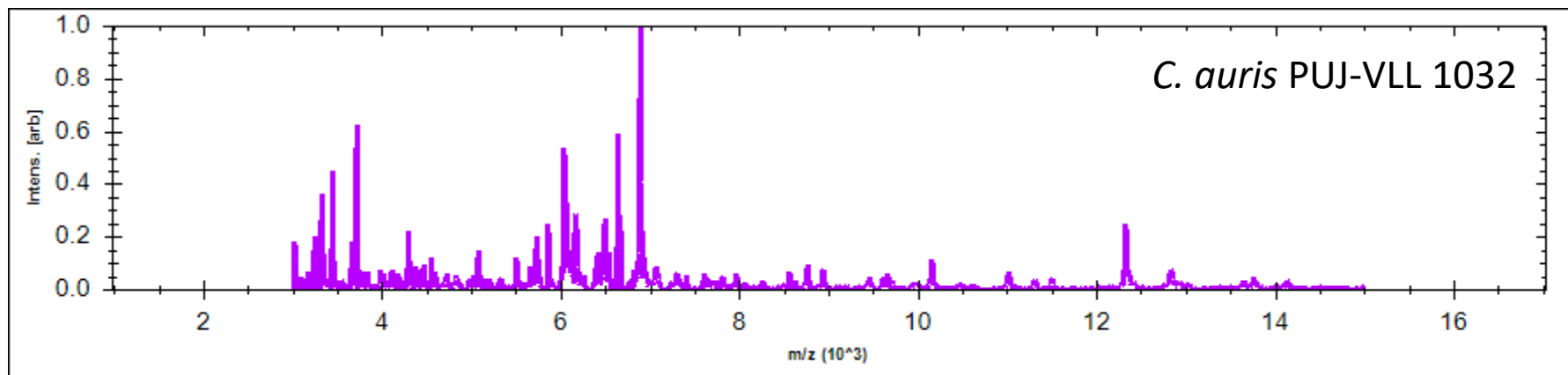

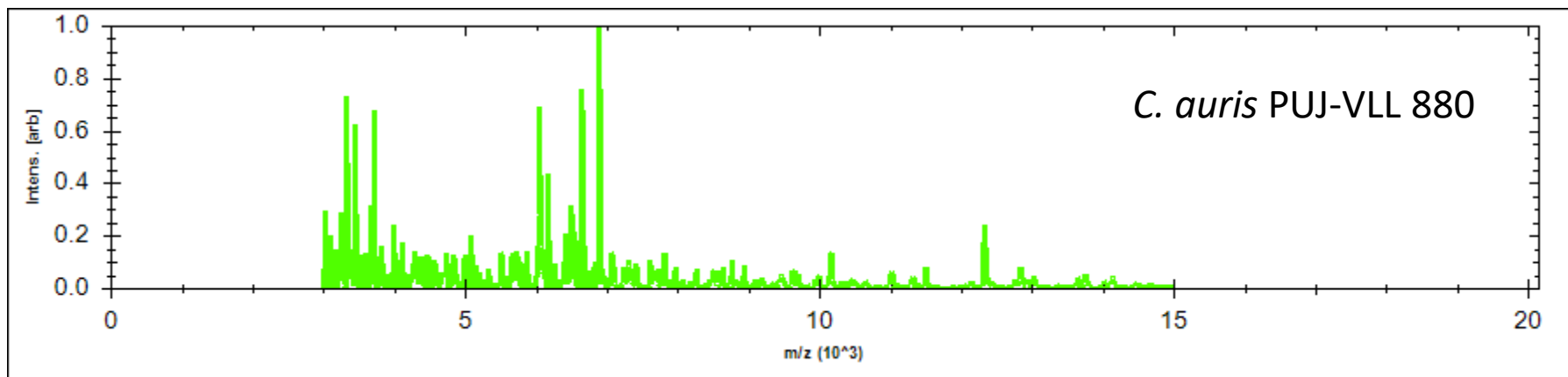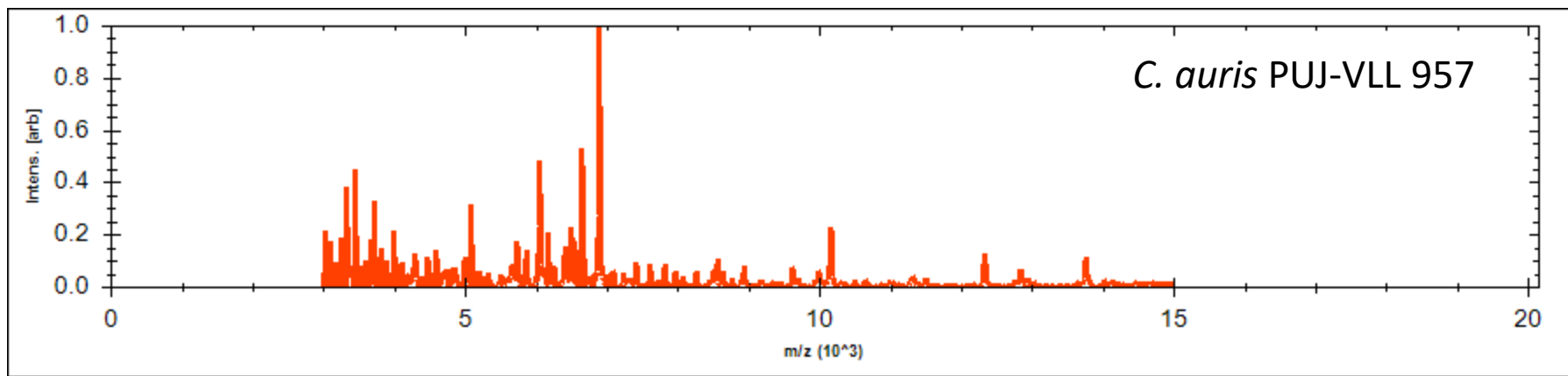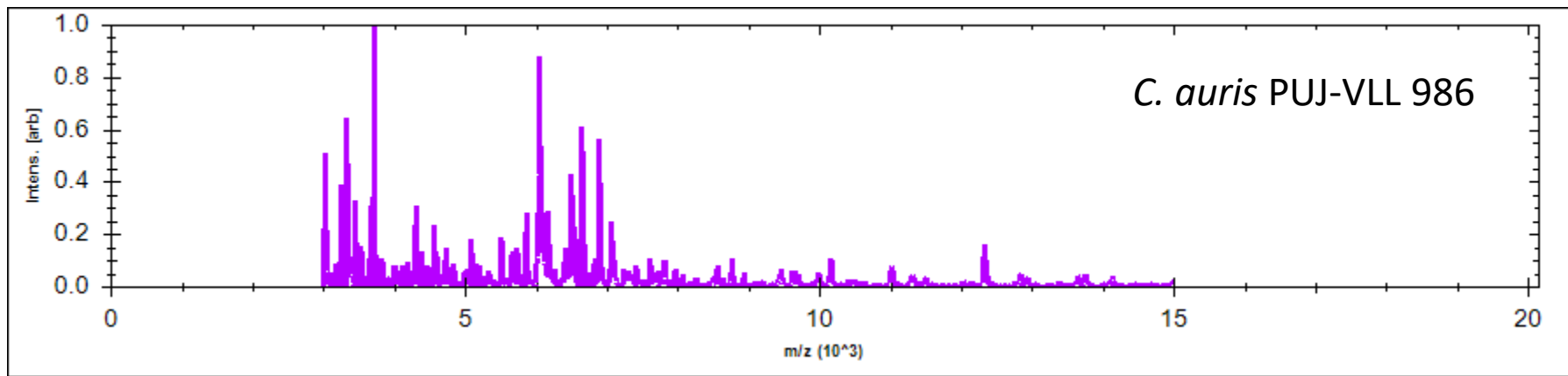

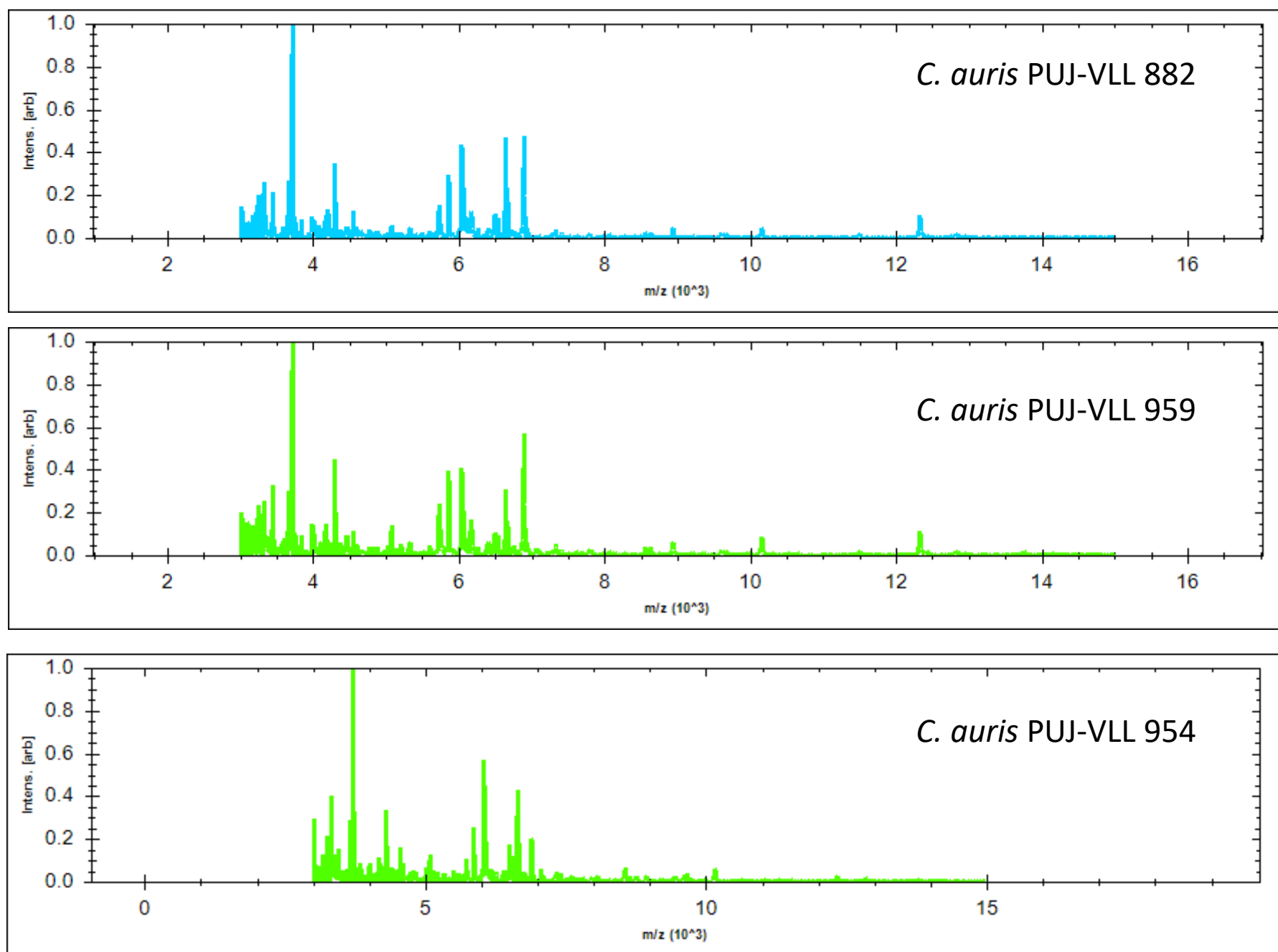

Figure S1. Mass Spectra from the strains used in Colombia library creation

Supplement: Supplementary file 1 [file jof-06-00072-s001.pdf]
